# Distinguishing Epileptiform Discharges From Normal Electroencephalograms Using Adaptive Fractal and Network Analysis: A Clinical Perspective

**DOI:** 10.3389/fphys.2020.00828

**Published:** 2020-08-05

**Authors:** Qiong Li, Jianbo Gao, Ziwen Zhang, Qi Huang, Yuan Wu, Bo Xu

**Affiliations:** ^1^School of Computer, Electronics and Information, Guangxi University, Nanning, China; ^2^Center for Geodata and Analysis, Faculty of Geographical Science, Beijing Normal University, Beijing, China; ^3^Institute of Automation, Chinese Academy of Sciences, Beijing, China; ^4^International College, Guangxi University, Nanning, Guangxi, China; ^5^The First Affiliated Hospital of Guangxi Medical University, Nanning, China

**Keywords:** EEG, epileptiform discharges, adaptive fractal analysis, Hurst parameter, singular value decomposition, brain network

## Abstract

Epilepsy is one of the most common disorders of the brain. Clinically, to corroborate an epileptic seizure-like symptom and to find the seizure localization, electroencephalogram (EEG) data are often visually examined by a clinical doctor to detect the presence of epileptiform discharges. Epileptiform discharges are transient waveforms lasting for several tens to hundreds of milliseconds and are mainly divided into seven types. It is important to develop systematic approaches to accurately distinguish these waveforms from normal control ones. This is a difficult task if one wishes to develop first principle rather than black-box based approaches, since clinically used scalp EEGs usually contain a lot of noise and artifacts. To solve this problem, we analyzed 640 multi-channel EEG segments, each 4*s* long. Among these segments, 540 are short epileptiform discharges, and 100 are from healthy controls. We have proposed two approaches for distinguishing epileptiform discharges from normal EEGs. The first method is based on Signal Range and EEGs' long range correlation properties characterized by the Hurst parameter *H* extracted by applying adaptive fractal analysis (AFA), which can also maximally suppress the effects of noise and various kinds of artifacts. Our second method is based on networks constructed from three aspects of the scalp EEG signals, the Signal Range, the energy of the alpha wave component, and EEG's long range correlation properties. The networks are further analyzed using singular value decomposition (SVD). The square of the first singular value from SVD is used to construct features to distinguish epileptiform discharges from normal controls. Using Random Forest Classifier (RF), our approaches can achieve very high accuracy in distinguishing epileptiform discharges from normal control ones, and thus are very promising to be used clinically. The network-based approach is also used to infer the localizations of each type of epileptiform discharges, and it is found that the sub-networks representing the most likely location of each type of epileptiform discharges are different among the seven types of epileptiform discharges.

## 1. Introduction

Epilepsy is a chronic neurological disease characterized by the paroxysmal seizures that affects people of all ages (Li et al., 2019). According to the WHO, about 50 million people worldwide have epilepsy, making it one of the most common neurological diseases in the world (Perkins, 2019). The ictal EEG is characterized by the presence of epileptiform discharges occurring before or after a seizure (Tautan et al., 2018). Unlike 24 h monitoring where one may be able to record the occurrence of seizures of a patient once or a few times, in clinical examination where only a few hours recording is considered feasible, often epileptiforms rather than actual seizures may be more likely to be observed. As epileptiform discharges can already provide information about seizure localization (Richards et al., 2018) and epileptic syndrome (Basiri et al., 2019), identification of epileptiform discharges is very important.

There are a variety of ways to represent EEG. Among the simplest and most popular are to compute the amplitude values (Toet et al., 2005), compute the Power Spectral Density (PSD) (Gao et al., 2007), or take wavelet transform (Adeli et al., 2003; Subasi, 2007; Faust et al., 2015; Chen et al., 2017). Clinically, however, neurologists still customarily examine the long continuous signals visually to identify epileptiform discharges or other features from EEG. Unfortunately, this is quite time-consuming and potentially inaccurate due to human fatigue. This problem has motivated much effort to develop novel algorithms to automatically detect epileptiform discharges or other features from EEG (Sharmila and Geethanjali, 2019). Among the notable works along this line are to use entropy (Nicolaou and Georgiou, 2012; Arunkumar et al., 2016, 2017) and complexity measures (Gao et al., 2011a, 2012b; Martis et al., 2015; Medvedeva et al., 2016; Pratiher et al., 2016; Sikdar et al., 2018). However, the majority of the works published are based on electrocorticogram (ECoG), which is invasively obtained by means of electrodes applied directly over or inserted into the cerebral cortex (Wang et al., 2019). Clinically, the more widely available form of EEG is the non-invasive surface EEG. Compared with ECoG, surface EEG signals are much poorer in terms of signal-to-noise ratios (Haufe et al., 2018). Besides noise, surface EEG recordings are also often contaminated by various kinds of artifacts (Islam et al., 2016; Brienza et al., 2019), including eye movements (e.g., blinking), muscle activities (e.g., swallowing, head movements), and the heartbeat (Kappel et al., 2017). These noise and artifacts greatly hinder the proper interpretation of the underlying neural information processing and add enormous difficulty to automatically identify epileptiform discharges from normal controls. Although machine learning based approaches (Mirowski et al., 2008; Shen et al., 2009; Antoniades et al., 2016; Kuswanto et al., 2017; Ullah et al., 2018; van Putten et al., 2018; Subasi et al., 2019) can partly solve some of these problems, overall, the problem remains to be challenging, and calls for easily-interpretable, less black-box based approaches.

To develop accurate fundamental principle-based instead of black-box based approaches to automatically detect epileptiform discharges, it is critical to comprehensively account for all the major features in the EEG that distinguish epileptiform discharges from normal ones. Based on this rationale, we will consider the long range correlation properties of EEG, together with the Signal Range and the relative energy in the alpha wave band of an EEG signal. The long range correlation properties are characterized by the Hurst parameter *H* which has been found to be able to characterize effectively dynamical changes in EEG signals. *H* is among the simplest measures from nonlinear science (Gao et al., 2007). Here we will employ adaptive fractal analysis (AFA) to compute *H* (Hu et al., 2009; Gao et al., 2011b, 2012a; Tung et al., 2011; Riley et al., 2012; Kuznetsov et al., 2013), which is an improvement DFA and can better deal with noise, non-stationarity, and various kinds of artifacts in surface EEG (Peng et al., 1994; Hu et al., 2001; Chen et al., 2002, 2005; Xu et al., 2005, 2011; Ma et al., 2010).

The human brain is comprised of numerous neurons that form a complicated network (Bashan et al., 2012; Bartsch et al., 2015; Liu et al., 2015; Ivanov et al., 2016; Denève et al., 2017; Gupta et al., 2018; Xue and Bogdan, 2019). Over the recent years, many researches have been conducted to elucidate the characteristics of cerebral network based on structural and functional scales (Smitha et al., 2017; Smith and Escudero, 2017; Xue and Bogdan, 2017; Gupta et al., 2018, 2019; Wang et al., 2018). The information yielded by an EEG channel is essentially the difference of electrical activity between two electrodes in the time-domain (Pardey et al., 1996; Lopez et al., 2016); the amplitude, frequency, and synchronization of the brain waves and background will change (Seeck et al., 2017; Vanherpe and Schrooten, 2017), depending on which montage is chosen (e.g., earlobe reference, averaged reference, or bipolar Christodoulakis et al., 2013; Geier and Lehnertz, 2017; Rana et al., 2017; Acharya and Acharya, 2019; Rios et al., 2019). For the EEG signals to reflect the networked nature of the brain, it is important to construct networks based on the EEG signals or the features of EEG. As we will discuss later, such a strategy has additional advantages in further suppressing noise and artifacts, and making the dependence of the results on the chosen montages weaker.

The remainder of the paper is organized as follows. In section 2, we briefly describe the EEG data and analysis methods. In section 3, we present results of our analysis. In section 4, we summarize our findings.

## 2. Materials and Methods

### 2.1. Data

The EEG data analyzed in this study were from the First Affiliated Hospital to Guangxi Medical University. The studies involving human participants were reviewed and approved by the ethics committee of the First Affiliated Hospital to Guangxi Medical University. The participants provided their written informed consent to participate in this study. Fifty-nine epilepsy patients underwent a 3 h video-EEG monitoring with 19-channel EEG recording with electrodes placed on the scalp under the international 10–20 system at 256 Hz sampling rate. The electrode impedances were kept below 10*K*Ω. The 19 scalp electroencephalographic electrodes were arranged according to the names *Fp*1, *Fp*2, *F*7, *F*3, *Fz*, *F*4, *F*8, *T*3, *C*3, *Cz*, *C*4, *T*4, *T*5, *P*3, *Pz*, *P*4, *T*6, *O*1, and *O*2.

**Figure 1:**
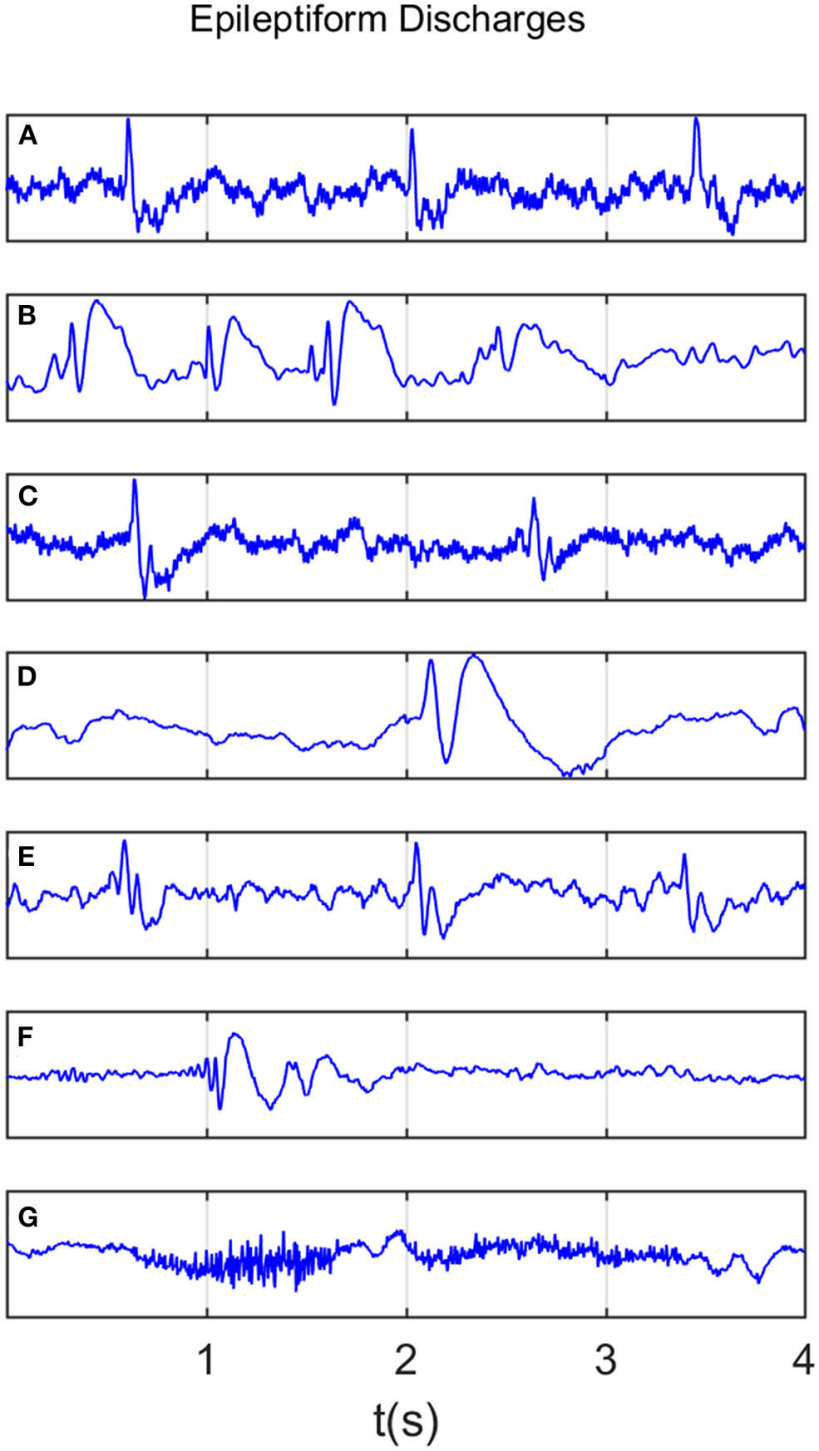
Typical waveforms of the 7 major epileptiform EEG, where **(A–G)**, denotes spike wave, spike and slow wave complex, sharp wave, sharp and slow wave complex, polyspike complex, polyspike and slow wave complex, spike rhythm discharges, respectively.

All epileptiform discharges were annotated by an experienced clinical neurophysiologist based on the average montage with an analog bandwidth of 0.1 ~ 70 *Hz* and a notch filter of 50*Hz*. EEG signals were segmented into 4*s* epochs and were assigned random numbers for each participant. The collected epochs were transformed into European Data Format (EDF) for further analysis. In total, there were 532 EEG recordings of epileptiform discharges and 100 healthy controls, each 4*s* long, from all the participants. Among the 532 short epileptic discharges, there were 69 spikes, 82 sharps, 174 spike, and slow wave complexes, 72 sharp and slow wave complexes, 64 polyspike complexes, 77 polyspike, and slow wave complexes and 2 spike rhythmic discharges. Note the numbers for these 7 epileptiform discharges sum up to 540, which is slightly larger than 532. The reason is a few discharges were considered to simultaneously belong to more than 1 of the 7 different epileptiform discharges. For convenience of referencing, the definitions for these 7 epileptiform discharges are listed below. Examples of their waveforms are shown in Figure 1.

- Spike: The spikes are the most basic paroxysmal EEG activity, with a duration of 20~70 *ms*. Amplitude varies but are typically >50 *uV* (Kane et al., 2017).
- Sharp: A sharp wave is similar to the spike, and its time limit is 70~200 *ms* (5~14 *Hz*). Its amplitude is between 100 and 200 *uV*, and the phase is usually negative.
- Spike and slow wave complex: An epileptiform pattern consisting of a spike and an associated slow wave following the spike, which can be clearly distinguished from the background activity; may be single or multiple (Kane et al., 2017).
- Sharp and slow wave complex: An epileptiform pattern consisting of a sharp wave and an associated slow wave following the sharp wave, which can be clearly distinguished from the background activity; may be single or multiple (Kane et al., 2017).
- Polyspike complex: A sequence of two or more spikes.
- Polyspike and slow wave complex: An epileptiform pattern consisting of two or more spikes associated with one or more slow waves.
- Spike rhythm: refers to a widespread 10~25 *Hz* spike rhythm outbreak, with an amplitude of 100~200 *uV* and the highest voltage in the frontal area, lasting more than 1 *s*.

Recall that a few epileptiform discharge waveforms were considered to simultaneously belong to more than 1 of the 7 different epileptiform discharges. Because of this, further considering the differences among the seven epileptiform discharges becomes impossible and is not pursued here.

### 2.2. Computation of the Signal Range and the Energy of the Alpha Wave Component

Often EEG epileptic discharges are associated with a larger amplitude than the normal control EEG. This motivates us to compute a simple statistic, which we call Signal Range, to quantify this effect. It is computed as follows:

(1) $$\begin{array}{l} Signal\;Range=\text{Maximum of }\left\{x\left(t\right),t\in \left[t_{1},t_{2}\right]\right\}\\ \;-\text{Minimum of }\left\{x\left(t\right),t\in \left[t_{1},t_{2}\right]\right\} \end{array}$$

where *x*(*t*) is the EEG signal. This procedure is applied to each of the 19 EEG signals with reference to the earlobes (i.e., the difference of the EEG signals measured at the 19 electrodes and the earlobes), or to the difference of the EEG signals according to the network construction, as detailed in section 2.4. In the former case (i.e., with reference to the earlobe), the final Signal Range is estimated as the average of the 10 largest Signal Range estimated from the 19 EEG signals.

In clinical applications, the brain wave is often categorized into five bands: delta (0.5~3 *Hz*), theta (4~7 *Hz*), alpha (8~13 *Hz*), beta (14~30 *Hz*), and gamma (>30 *Hz*), respectively. The alpha wave is most visible when human beings are relaxed with eyes closed. We have found that the alpha wave component on occipital area is often larger for epileptiform discharges. To compute this component, we employ a Fourier transform of the EEG signal, obtain the power spectral density (PSD), and finally integrate the PSD curve over the alpha wave band.

### 2.3. Adaptive Fractal Analysis (AFA)

AFA utilizes an adaptive detrending algorithm to extract globally smooth trend signals from the data for a given time scale and then analyzes the scaling of the residuals to the fit as a function of the time scale (Hu et al., 2009; Tung et al., 2011). The main steps of AFA to estimate *H* are as follows:

Suppose starting from a stationary incremental process *x*(1), *x*(2), *x*(3),…, construct a random walk through the following equation:

(2) $$\begin{array}{l} u\left(n\right)=\sum_{k=1}^{n}\left(x\left(k\right)-\bar{x}\right),n=1,2,3,\ldots ,N \end{array}$$

where $\bar{x}$ is the mean of the process. Based on this random walk *u*(*n*), we wish to get a global trend *v*(*i*), *i* = 1, 2, …, *N* for any specific time scale *w*, where *N* is the length of the original time series. This is achieved by dividing the above random walk process into overlapped windows, where the size of each window *w* contains an odd number of samples, and adjacent windows overlap by (*w* + 1)/2 samples. The random walk process in each window is fitted by a polynomial of order *M*, and the polynomials in overlapped regions are combined to yield a single global trend. Typically M should be 1 or 2, a linear or quadratic function. The local fitting ensures that the global trend is optimal or close to optimal, as locally Taylor series expansion is used.

After we get the global trend *v*(*i*) of *u*(*i*) by the above method, the residual *u*(*i*) − *v*(*i*) can describe the fluctuation around the global trend. For fractal processes, the Hurst exponent *H* can be computed by the following equation,

(3) $$\begin{array}{l} F^{\left(2\right)}\left(w\right)={\left[\frac{1}{N}\sum_{i=1}^{N}{\left(u\left(i\right)-v\left(i\right)\right)}^{2}\right]}^{\frac{1}{2}}\sim w^{H} \end{array}$$

The above equation means by calculating the variance of the residual between the original random walk process and the fitted global trend under a varying window *w*, we can obtain a linear (or multiple linear) relation between log_2_ *F*(*w*) and log_2_ *w*.

**Figure 2:**
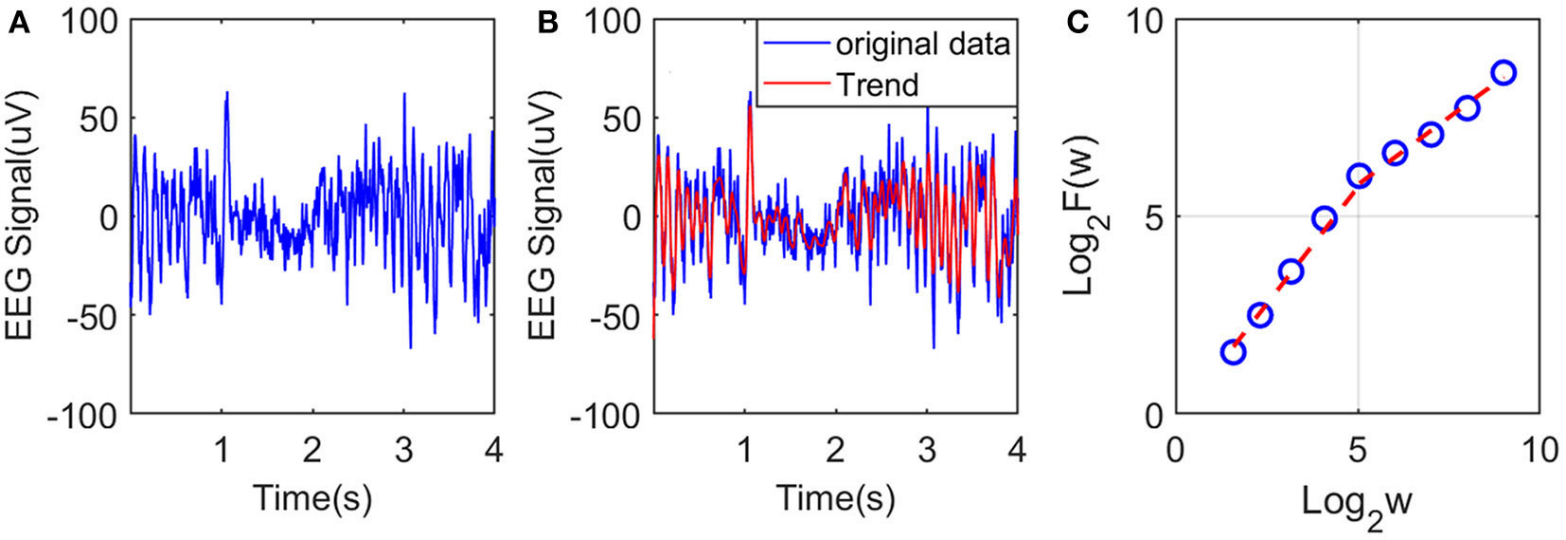
Illustration of estimation of the Hurst exponent using AFA: **(A)** raw EEG of a channel, **(B)** fitting (the red curve, with window size *w* = 21) of the EEG signal by the adaptive algorithm described, and **(C)** illustration of the scaling law with AFA.

To illustrate the procedures described, we have shown in Figure 2A an example of EEG signal and its global smooth trend in Figure 2B. By varying the window size *w*, we can obtain a curve of log_2_ *F*(*w*) and log_2_ *w* shown in Figure 2C, where we observe two scaling regimes, i.e., the curve can be fitted by two straight lines, with the slopes being the Hurst parameter on short and long time scales, respectively. The *H* on short time scales will be focused here.

### 2.4. Cerebral Network Construction

Brain activities involve spatial-temporal coordinated dynamics of numerous neurons in different regions of the brain, i.e., involve numerous functional brain networks. To better characterize the synergistic effects among the brain networks, it is important to construct brain networks based on multi-channel EEG signals. For this purpose, we consider networks with nodes being the 19 electrodes. Between any two of the nodes, we consider the difference between the two associated EEG signals. This is illustrated in Table 1 with a 19 × 19 table consisting of the difference of the EEG signals between two electrodes. Therefore, each element in the Table 1 is a time series. From it we can compute the Signal Range, the relative energy of the alpha wave component, and the Hurst parameter, as detailed earlier. Using each variable, we then obtain a network. Further analysis of these three networks will be based on singular value decomposition (SVD), which we will explain next.

**Table 1:** A 19 × 19 table consisting of the difference of the EEG signals between two electrodes.

Fp1-Fp1	Fp1-Fp2	Fp1-F7	Fp1-F3	Fp1-Fz	Fp1-F4	Fp1-F8	Fp1-T3	Fp1-C3	Fp1-Cz	Fp1-C4	Fp1-T4	Fp1-T5	Fp1-P3	Fp1-Pz	Fp1-P4	Fp1-T6	Fp1-O1	Fp1-O2
Fp2-Fp1	Fp2-Fp2	Fp2-F7	Fp2-F3	Fp2-Fz	Fp2-F4	Fp2-F8	Fp2-T3	Fp2-C3	Fp2-Cz	Fp2-C4	Fp2-T4	Fp2-T5	Fp2-P3	Fp2-Pz	Fp2-P4	Fp2-T6	Fp2-O1	Fp2-O2
F7-Fp1	F7-Fp2	F7-F7	F7-F3	F7-Fz	F7-F4	F7-F8	F7-T3	F7-C3	F7-Cz	F7-C4	F7-T4	F7-T5	F7-P3	F7-Pz	F7-P4	F7-T6	F7-O1	F7-O2
F3-Fp1	F3-Fp2	F3-F7	F3-F3	F3-Fz	F3-F4	F3-F8	F3-T3	F3-C3	F3-Cz	F7-C4	F3-T4	F3-T5	F3-P3	F3-Pz	F3-P4	F3-T6	F3-O1	F3-O2
Fz-Fp1	Fz-Fp2	Fz-F7	Fz-F3	Fz-Fz	Fz-F4	Fz-F8	Fz-T3	Fz-C3	Fz-Cz	Fz-C4	Fz-T4	Fz-T5	Fz-P3	Fz-Pz	Fz-P4	Fz-T6	Fz-O1	Fz-O2
F4-Fp1	F4-Fp2	F4-F7	F4-F3	F4-Fz	F4-F4	F4-F8	F4-T3	F4-C3	F4-Cz	F4-C4	F4-T4	F4-T5	F4-P3	F4-Pz	F4-P4	F4-T6	F4-O1	F4-O2
F8-Fp1	F8-Fp2	F8-F7	F8-F3	F8-Fz	F8-F4	F8-F8	F8-T3	F8-C3	F8-Cz	F8-C4	F8-T4	F8-T5	F8-P3	F8-Pz	F8-P4	F8-T6	F8-O1	F8-O2
T3-Fp1	T3-Fp2	T3-F7	T3-F3	T3-Fz	T3-F4	T3-F8	T3-T3	T3-C3	T3-Cz	T3-C4	T3-T4	T3-T5	T3-P3	T3-Pz	T3-P4	T3-T6	T3-O1	T3-O2
C3-Fp1	C3-Fp2	C3-F7	C3-F3	C3-Fz	C3-F4	C3-F8	C3-T3	C3-C3	C3-Cz	C3-C4	C3-T4	C3-T5	C3-P3	C3-Pz	C3-P4	C3-T6	C3-O1	C3-O2
Cz-Fp1	Cz-Fp2	Cz-F7	Cz-F3	Cz-Fz	Cz-F4	Cz-F8	Cz-T3	Cz-C3	Cz-Cz	Cz-C4	Cz-T4	Cz-T5	Cz-P3	Cz-Pz	Cz-P4	Cz-T6	Cz-O1	Cz-O2
T4-Fp1	T4-Fp2	T4-F7	T4-F3	T4-Fz	T4-F4	T4-F8	T4-T3	T4-C3	T4-Cz	T4-C4	T4-T4	T4-T5	T4-P3	T4-Pz	T4-P4	T4-T6	T4-O1	T4-O2
T5-Fp1	T5-Fp2	T5-F7	T5-F3	T5-Fz	T5-F4	T5-F8	T5-T3	T5-C3	T5-Cz	T5-C4	T5-T4	T5-T5	T5-P3	T5-Pz	T5-P4	T5-T6	T5-O1	T5-O2
P3-Fp1	P3-Fp2	P3-F7	P3-F3	P3-Fz	P3-F4	P3-F8	P3-T3	P3-C3	P3-Cz	P3-C4	P3-T4	P3-T5	P3-P3	P3-Pz	P3-P4	P3-T6	P3-O1	P3-O2
Pz-Fp1	Pz-Fp2	Pz-F7	Pz-F3	Pz-Fz	Pz-F4	Pz-F8	Pz-T3	Pz-C3	Pz-Cz	Pz-C4	Pz-T4	Pz-T5	Pz-P3	Pz-Pz	Pz-P4	Pz-T6	Pz-O1	Pz-O2
P4-Fp1	P4-Fp2	P4-F7	P4-F3	P4-Fz	P4-F4	P4-F8	P4-T3	P4-C3	P4-Cz	P4-C4	P4-T4	P4-T5	P4-P3	P4-Pz	P4-P4	P4-T6	P4-O1	P4-O2
T6-Fp1	T6-Fp2	T6-F7	T6-F3	T6-Fz	T6-F4	T6-F8	T6-T3	T6-C3	T6-Cz	T6-C4	T6-T4	T6-T5	T6-P3	T6-Pz	T6-P4	T6-T6	T6-O1	T6-O2
O1-Fp1	O1-Fp2	O1-F7	O1-F3	O1-Fz	O1-F4	O1-F8	O1-T3	O1-C3	O1-Cz	O1-C4	O1-T4	O1-T5	O1-P3	O1-Pz	O1-P4	O1-T6	O1-O1	O1-O2
O2-Fp1	O2-Fp2	O2-F7	O2-F3	O2-Fz	O2-F4	O2-F8	O2-T3	O2-C3	O2-Cz	O2-C4	O2-T4	O2-T5	O2-P3	O2-Pz	O2-P4	O2-T6	O2-O1	O2-O2

### 2.5. Singular Value Decomposition (SVD)

SVD is a decomposition method that can be applied to arbitrary matrices. For an *n* × *m* matrix *A*, it is generally expressed as:

(4) $$\begin{array}{l} A=U\Sigma V^{T} \end{array}$$

where, *U*_*n*×*n*_ and *V*_*m*×*m*_ are orthogonal matrices, which are composed of eigenvectors of square matrices, *AA*^*T*^ and *A*^*T*^*A*, respectively. Σ_*n*×*m*_, called the singular value matrix, is non-zero only on the main diagonal with the elements there being the square root of the eigenvalues of *AA*^*T*^ (or *A*^*T*^*A*). Denote them by Σ_*ii*_ = σ_*i*_, *i* = 1, 2, …, *r*, where *r* is the rank of *AA*^*T*^ (or *A*^*T*^*A*). They are usually written in descending order. In this work, we only need the largest singular value of the three networks based on the Signal Range, the energy of the alpha wave component, and the Hurst parameter.

### 2.6. Inference of the Localization of the Epileptiform Discharges

Based on the networks constructed using the three variables, the signal range, the relative energy of the alpha wave component, and the Hurst parameter, and using SVD, we can infer the localization of each type of epileptiform discharges. The approach is as follows. For each network of a subject, after we obtain the SVD, we project each column vector of the network to the singular vector corresponding to the largest singular value. The vector is then retained if the absolute value of the projection coefficient is ≥ 0.5. These vectors allow us to determine which channels of the original data are important. The procedure is applied to each of the three networks of the subject. We assume the common channels indicate the localization of this particular type of epileptiform discharge for that subject. As this localization may vary among subjects, we determine the most likely localization of a particular type of epileptiform discharge for all relevant subjects by requiring that each channel occurs at least with certain probability. Here, we has chosen this probability to be 0.55.

### 2.7. Random Forest Classifier (RF)

Random forest (RF) is an ensemble-based learning technique for classification (Cutler et al., 2012), which has been shown to have high accuracy, is not affected by overtraining, and does not require normalization of the input data. It consists of many separate classification trees, each of which is obtained through a separate bootstrap sample from the data set and each tree classifies the data. A majority vote among the trees provides the final result.

The objective of the RF classifiers used here is to classify which of the two classes an EEG signal belongs to: normal or epileptic discharges. The inputs to the RF classifier are the square of the largest singular values of the three networks (e.g., based on the Signal Range, the energy of the alpha wave component, and Hurst parameters) based on SVD. Following usual practice, we have randomly taken one-third of the total data as testing data and two-thirds of the data for training the model in this paper.

### 2.8. Evaluation of Performance

To assess the consistency of the diagnosis by the neurologists and machine classification, we need to compute the classification accuracy. This can be accomplished by computing the receiver operating characteristic (ROC) curve and many statistics derived from the ROC curve. In fact, all these are best understand with the confusion matrix, which is a table with two rows and two columns that reports the number of false positives (FP), false negatives (FN), true positives (TP), and true negatives (TN). From them we can define three major metrics:

(5) $$\begin{array}{l} sensitivity=\frac{TP}{TP+FN} \end{array}$$

(6) $$\begin{array}{l} specificity=\frac{TN}{TN+FP} \end{array}$$

(7) $$\begin{array}{l} accuracy=\frac{TP+TN}{TP+FP+TN+FN} \end{array}$$

Note that the sensitivity is also called true positive rate (TPR) and 1 − *specificity* is also called false positive rate (FPR).

The ROC is a plot of TPR vs. FPR using different threshold values as a sweeping variable. Not suffering from class imbalance, the ROC is a good way to characterize imbalanced data sets. The area below the ROC is called area under curve (AUC). Its value takes from 0 to 1. A value of AUC being 0.5 means the classification model has no predictive ability at all. On the other hand, when the value of AUC reaches 1, it means that the probability density functions of negative and positive classes are completely separated, and the prediction ability is 100%. This is equivalent to the ROC being a unit step function.

## 3. Results

**Figure 3:**
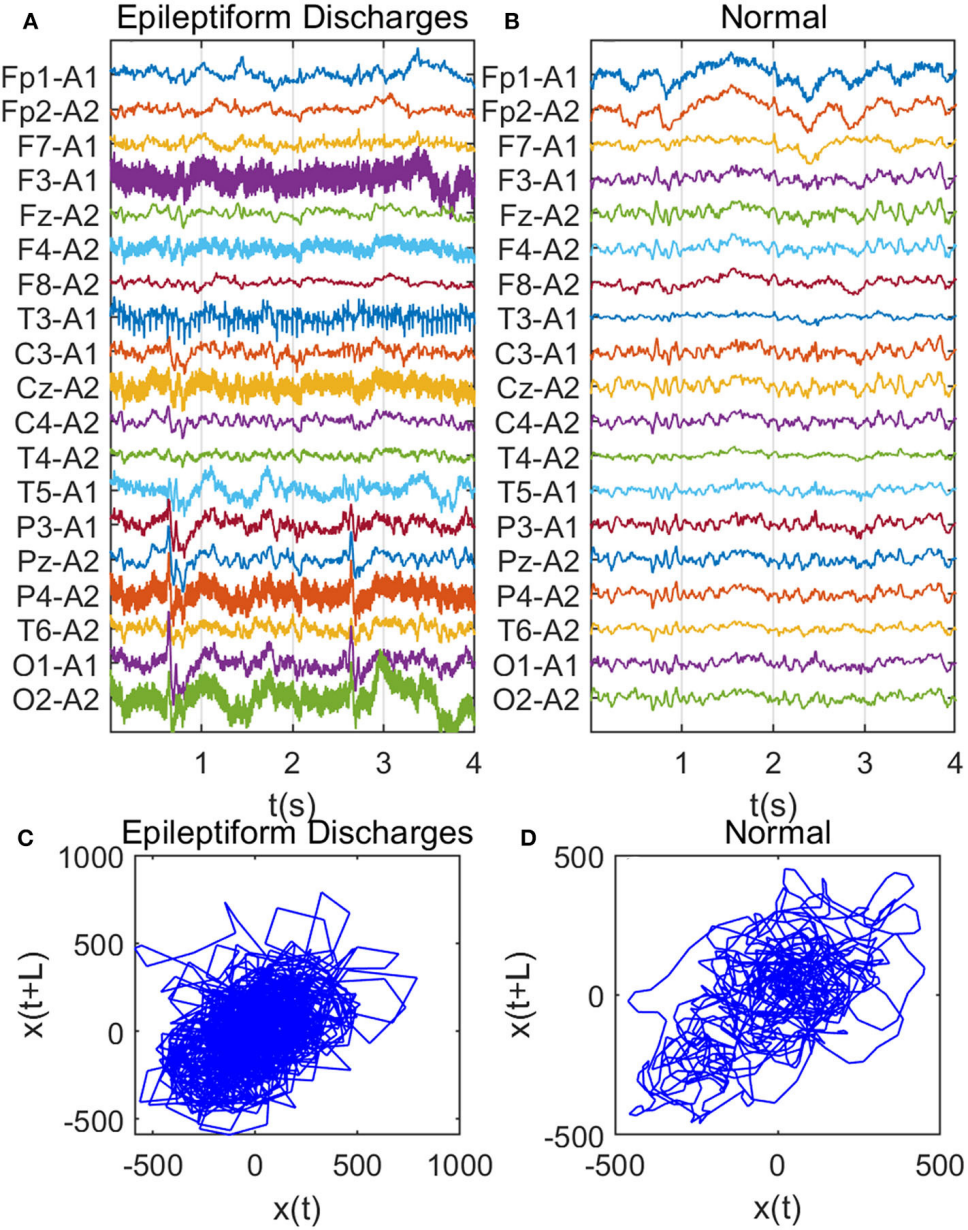
Comparison of epileptiform discharges and normal EEG: **(A)** example of epileptic discharges, **(B)** normal EEG, **(C,D)** 2-D phase diagrams using the summation of the 19 epileptiform discharges and normal EEG signals shown in **(A,B)**, respectively, which can be used to estimate Signal Range.

**Figure 4:**
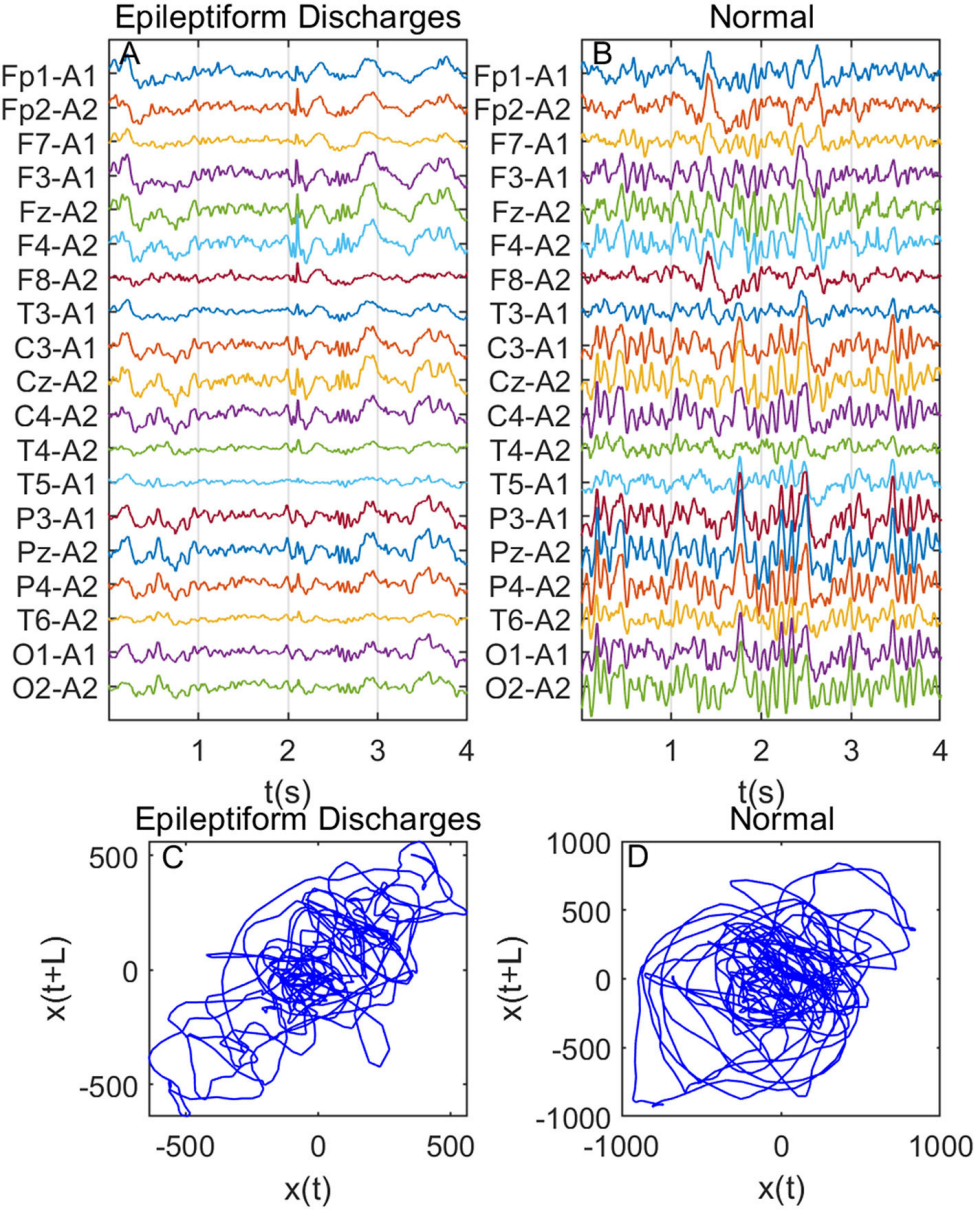
Same as Figure 3, except data were from another subject showing that Signal Range for epileptiform discharges can be smaller than that of normal EEG: **(A)** example of epileptic discharges, **(B)** normal EEG, **(C,D)** 2-D phase diagrams using the summation of the 19 epileptiform discharges and normal EEG signals shown in **(A,B)**, respectively, which can be used to estimate Signal Range.

Recall that among the 640 EEG data sets analyzed here, 69, 82, 174, 72, 64, 77, and 2 data sets are for spike, sharp, spike and slow wave complex, sharp and slow wave complex, polyspike complex, polyspike and slow wave complex, and spike rhythm, respectively, and 100 are for normal controls. Figures 3A,B depicts examples of typical wave forms of epileptiform discharge and the normal EEG. One easy way to appreciate their difference is to construct 2-D phase diagrams shown in Figures 3C,D, which are constructed using the summation of the 19 EEG signals shown in Figures 3A,B. As one can easily understand, the Signal Range can be conveniently estimated from such 2-D phase diagrams. On average, we have observed that the Signal Range is larger for epileptiform discharges than for normal controls. However, this is only in terms of average. Opposite situations also exist. An example is shown in Figure 4, where we observe that the Signal Range for epileptiform discharges can be much smaller than that of normal EEG. Of course, such cases are well-known in the literature and clinically, and motivate us to also account for other features of EEG signals.

**Figure 5:**
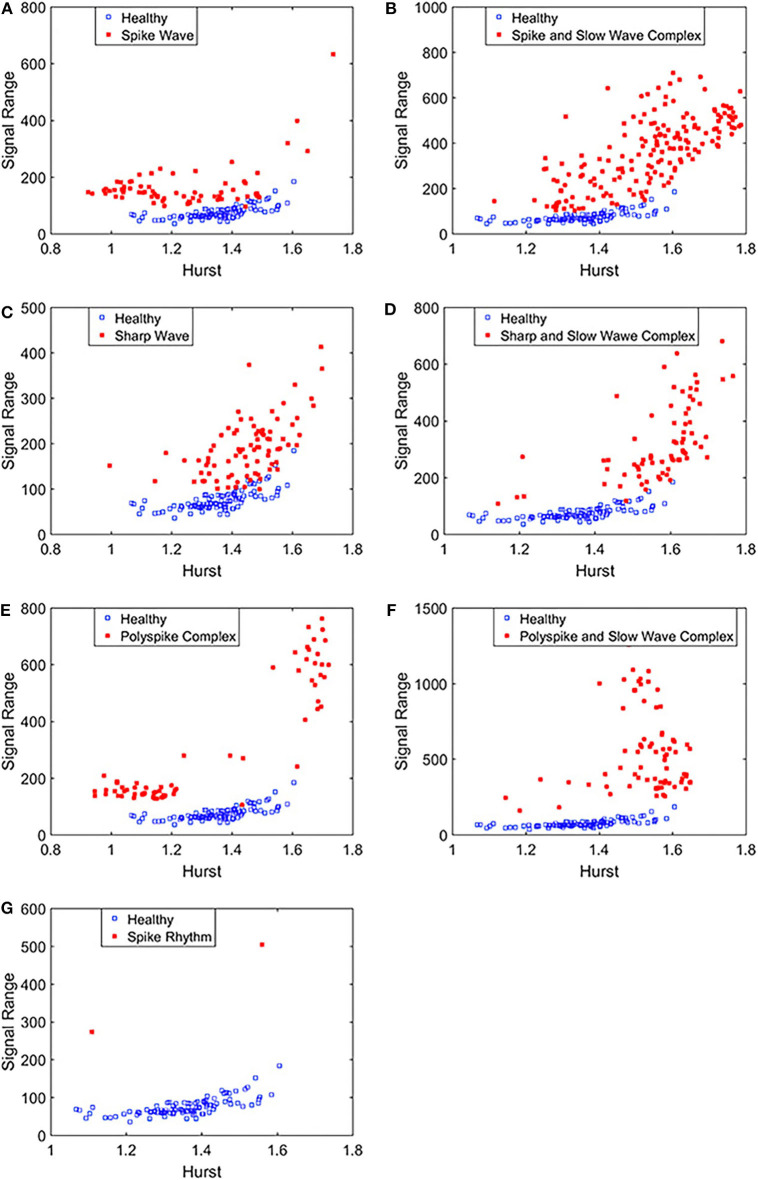
Scatter plots using features Signal Range and the Hurst parameter *H*, where **(A–G)**, illustrates the different between the seven types of epileptiform discharges (spike wave, spike and slow wave complex, sharp wave, sharp and slow wave complex, polyspike complex, polyspike and slow wave complex, spike rhythm discharges) and normal EEG. These plots highly suggest the classification accuracy will be very high.

To complement the Signal Range, let us examine the long range correlations captured by the Hurst parameter *H*. We have calculated *H* for the 19 EEG signals shown in Figures 3, 4 and then taken the average. In Figure 5, we have constructed scatter plots using Signal Range and Hurst parameter *H*. We observe that the three cases, the polyspike and slow wave complex and the spike rhythm, are completely separated from the normal control group, as shown in Figures 5F,G. The separations for the other 5 cases, although not 100%, are also quite good, as is evident from Figures 5A–E. These plots highly suggest the classification accuracy will be very high.

**Figure 6:**
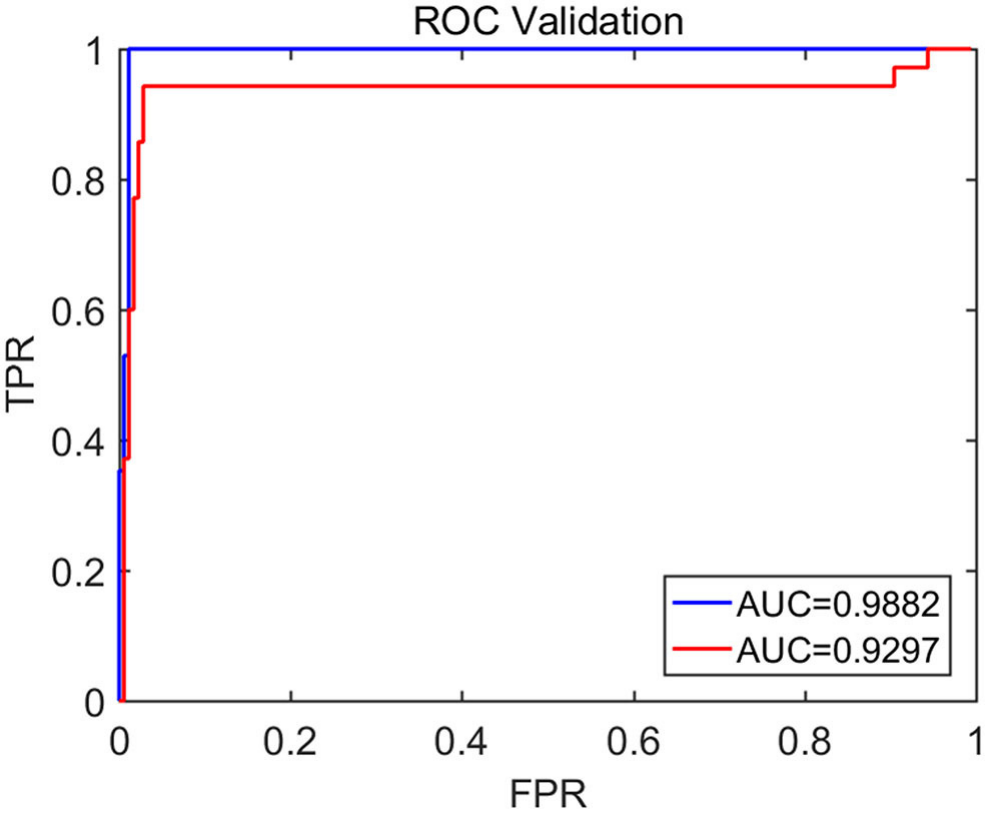
The ROC curve for the testing data. The red and blue curves show respectively the ROC based on methods using Signal Range and *H* and networks built on Signal Range, energy of the alpha wave component, and *H*. The AUC for the blue and red curves is 0.9882 and 0.9297, respectively.

To compute the classification accuracy based on the Signal Range and the Hurst parameter, we have employed the RF classifier. We have randomly taken two-thirds of the data as the training data and the remaining one-third of the total data as the testing data. The class distribution of the samples in the training and testing data set is summarized in Table 2. The test performance of the classifier can be determined by computing the metrics defined in section 2.7. The confusion matrix in Table 3 (Method One) shows that 6 out of 34 normal subjects are classified incorrectly by the RF as the epileptiform discharge, 5 out of 180 epileptiform discharges are classified incorrectly as the normal subject. Table 4 shows classification performance. It can be seen that it provides the accuracy of 94.86%, sensitivity and specificity of 97.22 and 82.35%. Figure 6 (the red curve) shows the ROC curve for the testing data of the RF classifier with all seven types of epileptiform discharges grouped into one super class. The AUC of the red curve is 0.9297.

**Table 2:** Class distribution of the samples in the training and test data sets.

**Classes**	**Training set**	**Testing set**	**Total**
Normal controls	66	34	100
Epileptiform discharges	360	180	540
Total	426	214	640

**Table 3:** Confusion Matrix for the testing data of 180 epileptiform discharges and 34 normal controls: Method One uses Signal Range and *H*, Method Two is based on the networks constructed from the Signal Range, the energy of the alpha wave component, and the *H*.

**Method**	**Result**	**Epileptiform discharges**	**Healthy controls**
Method one	Epileptiform discharges	175	5
	Healthy controls	6	28
Method two	Epileptiform discharges	178	2
	Healthy controls	1	33

**Table 4:** Classification performance measures.

**Method**	**Sensitivity (%)**	**Specificity (%)**	**Accuracy (%)**
Signal range and Hurst	97.22	82.35	94.86
The network based on Signal Range, alpha band energy, and *H*	98.89	97.06	98.60

**Figure 7:**
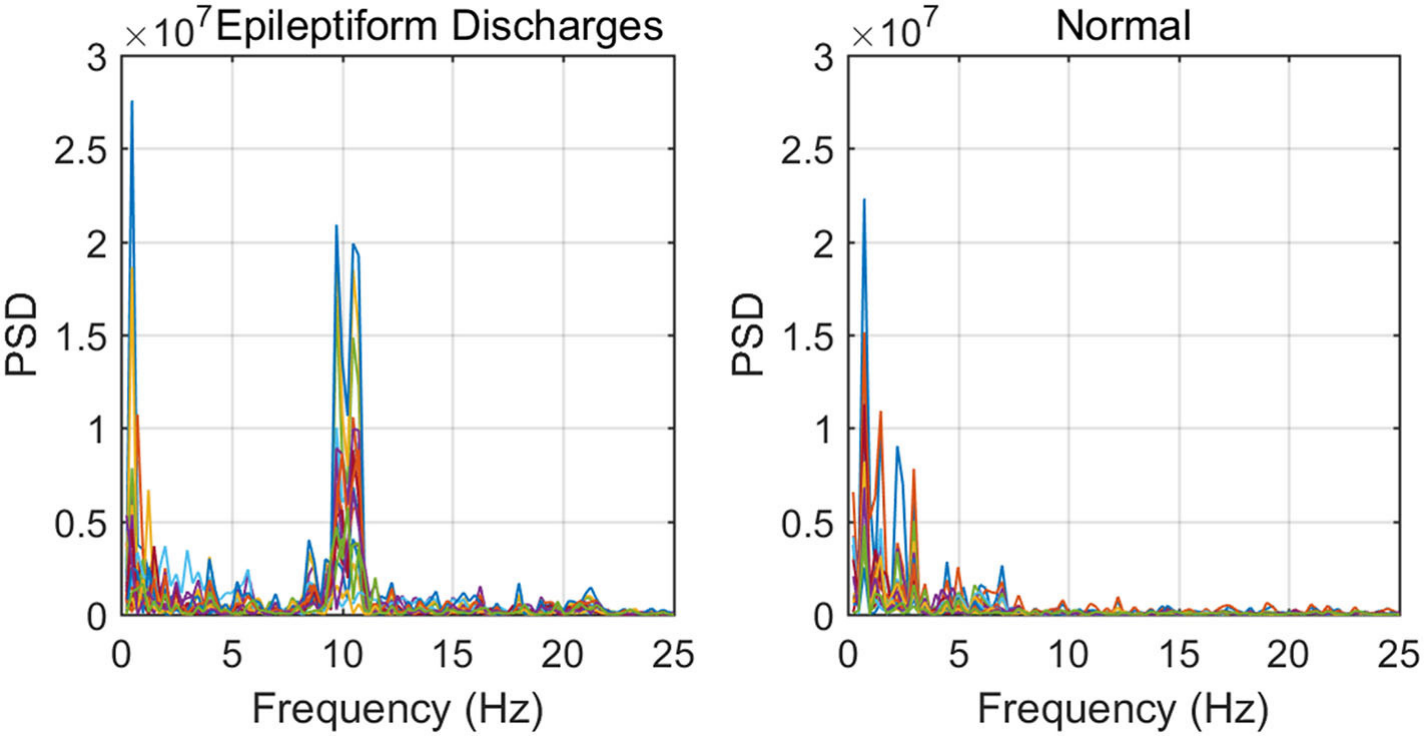
Typical PSD curves for epileptiform discharges and normal EEG showing that the relative energy of the alpha wave component for epileptiform discharges is often larger for that of normal EEGs.

**Figure 8:**
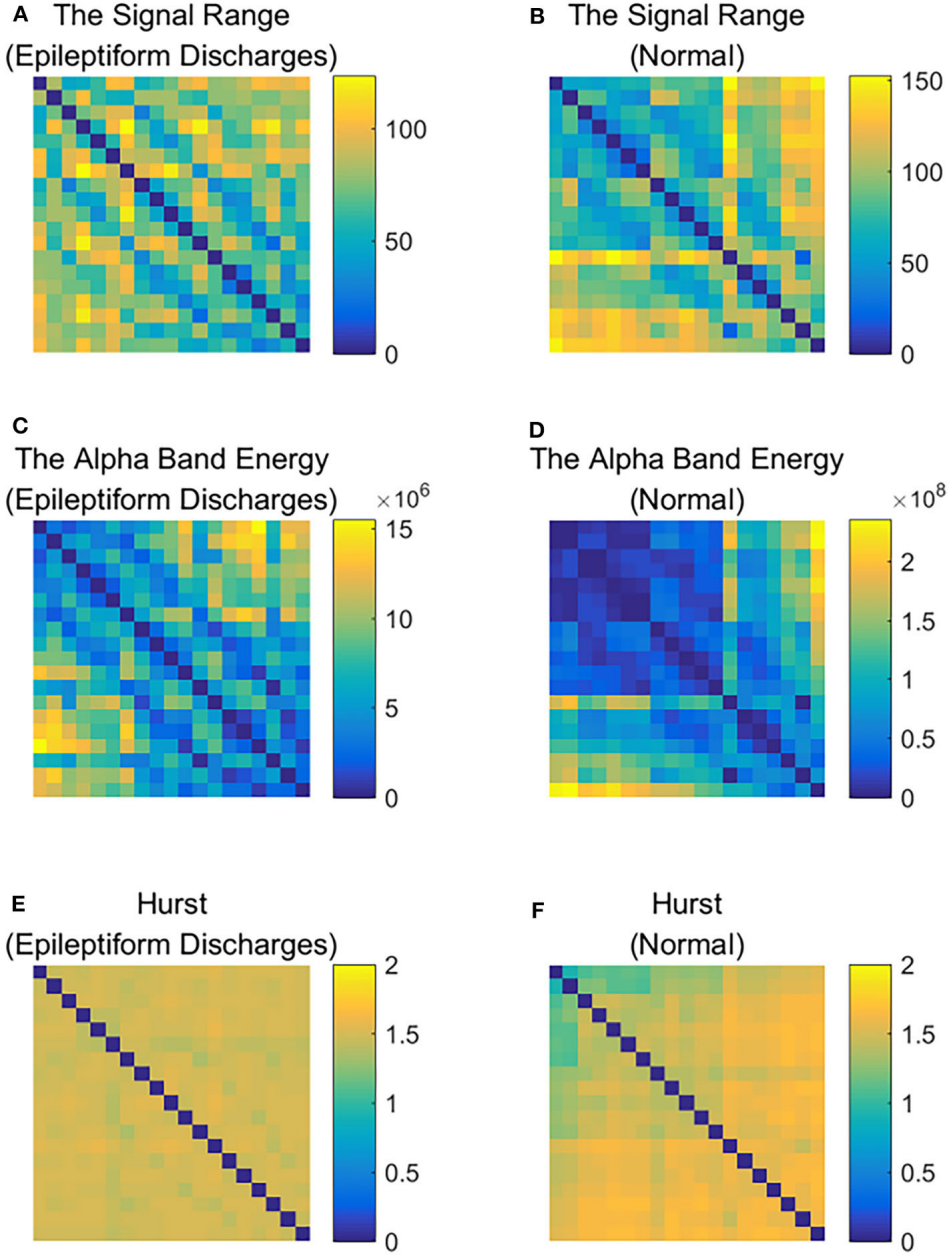
Heat maps illustrating the three types of networks described in section 2: **(A,C,E)** are for epileptiform discharges while **(B,D,F)** are for normal EEG.

**Figure 9:**
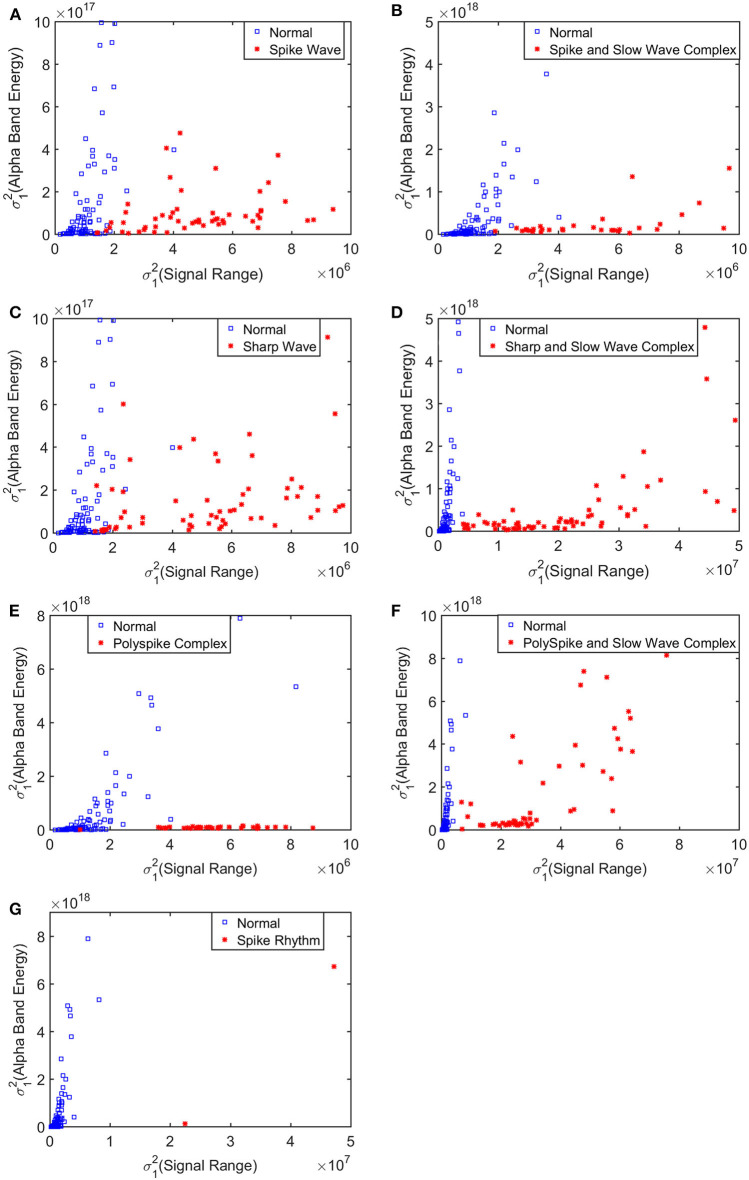
Scatter plots using features from networks based on the Hurst parameter and the Signal Range, where **(A–G)**, illustrates the different between the seven types of epileptiform discharges (spike wave, spike and slow wave complex, sharp wave, sharp and slow wave complex, polyspike complex, polyspike and slow wave complex, spike rhythm discharges) and normal EEG. These plots highly suggest the classification accuracy will be very high.

To improve the accuracy of classification, we have developed a brain network based approach. Specifically, three separate networks are constructed, based on the Signal Range, the energy of the alpha wave component, and *H*. Extracting the Signal Range is straightforward. Extracting the energy of the alpha wave component is a little more complicated, but can be readily done (Gao et al., 2007). As shown in Figure 7, we can see that typical PSD for epileptiform discharges and normal EEG show significant difference in the energy of the alpha wave component: it is often larger for epileptiform discharges than for normal. Obtaining *H* has already been done. Examples of heat maps for these networks are shown in Figure 8. Each of these networks is further analyzed by SVD. We have focused on the square of the first singular value as the final features. In Figure 9, we have constructed scatter plots using the square of the first singular values of the networks based on the Signal Range and the energy of the alpha wave component. We observe that the difference between the seven types of epileptiform discharges and the normal EEG is very significant.

Again, let us input the square of the first singular values of the networks based on the Signal Range, the energy of the alpha wave component, and the *H* to the RF classifier. Table 3 (Method Two) shows that 1 out of 34 normal subjects are classified incorrectly as the epileptiform discharge, while 2 out of 180 epileptiform discharge is classified incorrectly as the normal subject. Clearly, this network based method is much improved over the first method, which is based on Signal Range and the Hurst parameter, as the number of misclassifications with this new method is much reduced. With this network based method, the RF classifier has a sensitivity, specificity, and accuracy of 98.89, 97.06, and 98.60%, respectively, in contrast with that of 97.22, 82.35, and 94.86%, which are the basic parameters for the method based on the Signal Range and the Hurst parameter. These numbers are summarized in Table 4, and the blue ROC curve shown in Figure 6 (with all seven types of epileptiform discharges grouped into one super class). While the ROC curve is already close to a unit step function, the result for the training data is even better (and thus not shown here).

We have tried to infer the localizations of each type of epileptiform discharges based on the approach described in section 2.6, whose essence is to equate the sub-network representing the localization of each type of epileptiform discharge to the nodes which generate the most likely alpha band energy, signal range, and the Hurst parameter of that type of epileptiform discharge. The result is shown in Table 5. We observe that while the channels *O*1 and *O*2 have appeared in most of the epileptiform discharges, the sub-networks representing the most likely location of each type of epileptiform discharges are different among the seven types of epileptiform discharges studied here.

**Table 5:** The localization of the epileptiform discharges.

**Epileptiform discharges**	**The scape location**
Spike	*F*7; *F*4; *T*3; *T*4; *T*5; *O*1; *O*2
Spike and slow wave complex	*C*4; *T*4; *P*3; *P*4; *T*6; *O*2
Sharp	*T*3; *T*4; *P*3; *O*1; *O*2
Sharp and slow wave complex	*F*8; *T*3; *T*4; *T*6; *O*1; *O*2
Polyspike complex	*F*7; *F*4; *F*8; *T*3; *T*5; *T*6; *O*1; *O*2
Polyspike and slow wave complex	*Fp*1; *F*8; *T*3; *C*4; *T*4; *T*6; *O*1; *O*2
Spike rhythm	*Fp*2; *Fz*; *F*4; *T*3; *T*5; *Pz*

Finally, we have compared our results with that of Anh-Dao et al. (2018), who developed an expert system employing multiple state-of-the-art signal processing and machine learning techniques including wavelet transform, spectral filtering, and artificial neural networks for the purpose of automatically detecting epileptic spikes. They achieved an AUC of 0.945, which is slightly better than our Signal Range and the Hurst parameter based method. This is understandable, since our Signal Range and the Hurst parameter based method is so much simpler than their method. Interestingly, our network based approach, which is of similar simplicity with our Signal Range and the Hurst parameter based method, is much more accurate that their method, since our AUC is 0.9882. Most importantly, both of our methods are based on fundamental principles rather than the black-box approach, and therefore, either of our method has the prospect of being widely deployed in clinical setting.

## 4. Conclusion

In this paper, we have proposed two approaches for distinguishing epileptiform discharges from normal EEGs, with the aim of being able to use them widely in a clinical setting. Our first method is based on Signal Range and the Hurst parameter. Every component of our method can be readily understood and implemented based on first principles. Although simple, the approach already achieves a high detection accuracy of 94.86%. To improve the accuracy of detection, our second method employs the notion of network, with the hope of capturing the functioning of human brain network to some degree. Specifically, our approach involves three types of networks, one based on the Signal Range, the second based on the energy of the alpha wave component of EEG, and the third based on the Hurst parameter. Each of the networks is analyzed by SVD, and the square of the first singular value is utilized to construct features to distinguish epileptiform discharges from normal controls. This network based approach, while still fully first principle based and readily understandable, achieves a very high accuracy of 98.60%. This accuracy is higher than a recent approach proposed by Anh-Dao et al. (2018), which was an expert system employing multiple state-of-the-art signal processing and machine learning techniques including wavelet transform, spectral filtering, and artificial neural networks for the purpose of automatically detecting epileptic spikes. Most importantly, both of our methods are based on fundamental principles rather than the black-box approach, and therefore, are very promising to be used clinically.

We have also designed a network-based approach to infer the localizations of each type of epileptiform discharges based on the networks constructed using the three variables, the signal range, the relative energy of the alpha wave component, and the Hurst parameter. The essence of the approach is to equate the sub-network representing the localization of each type of epileptiform discharge to the nodes which generate the most likely alpha band energy, signal range, and the Hurst parameter of that type of epileptiform discharge. We have found that while the channels *O*1 and *O*2 have appeared in most of the epileptiform discharges, the sub-networks representing the most likely location of each type of epileptiform discharges are different among the seven types of epileptiform discharges studied here.

It is worth noting that the epileptiform discharges analyzed here were provided in two batches: in the first batch, which was about 2/3 of the data analyzed here, the accuracy was similar to that reported here. Then more epileptiform data were given to us by clinical doctors to examine whether the accuracy remained as high. It was yes. Nevertheless, the data analyzed here were still quite limited. It would be interesting and important to further validate the proposed approaches with more data in different clinical sets.

## Data Availability Statement

The raw data supporting the conclusions of this article will be made available by the authors, without undue reservation.

## Ethics Statement

The studies involving human participants were reviewed and approved by the ethics committee of the First Affiliated Hospital to Guangxi Medical University. The participants provided their written informed consent to participate in this study.

## Author Contributions

QL performed most of the experimental work. ZZ assisted in data analysis. QH and YW provided the data needed for this experiment and engaged in many analysis and discussions. BX engaged in many discussions. JG conceived the study, provided overall supervision for the study and directed all phases of the study and including writing of the manuscript. All authors read and approved the final manuscript.

## Conflict of Interest

The authors declare that the research was conducted in the absence of any commercial or financial relationships that could be construed as a potential conflict of interest.
